# Analysis of Coronary Vessels in Cleared Embryonic Hearts

**DOI:** 10.3791/54800

**Published:** 2016-12-07

**Authors:** Sarah Ivins, Catherine Roberts, Bertrand Vernay, Peter J. Scambler

**Affiliations:** ^1^Developmental Biology of Birth Defects, UCL Institute of Child Health; ^2^MRC Centre for Regenerative Medicine, SCRM Building, University of Edinburgh

**Keywords:** Developmental Biology, Issue 118, coronary vessels, BABB, PECAM1, embryonic heart, wholemount immunofluorescence, optical clearing, confocal microscopy

## Abstract

Whole mount visualization of the embryonic coronary plexus from which the capillary and arterial networks will form is rendered problematic using standard microscopy techniques, due to the scattering of imaging light by the thick heart tissue, as these vessels are localized deep within the walls of the developing heart. As optical clearing of tissues using organic solvents such as BABB (1 part benzyl alcohol to 2 parts benzyl benzoate) has been shown to greatly improve the optical penetration depth that can be achieved, we combined clearance of whole, PECAM1-immunostained hearts, with laser-scanning confocal microscopy, in order to obtain high-resolution images of vessels throughout the entire heart. BABB clearance of embryonic hearts takes place rapidly and also acts to preserve the fluorescent signal for several weeks; in addition, samples can be imaged multiple times without loss of signal. This straightforward method is also applicable to imaging other types of blood vessels in whole embryos.

**Figure:**
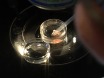


## Introduction

Establishing a functioning coronary network is crucial for heart function and embryonic development, and analysis of genetic mouse mutants can provide valuable insight into the molecular signals underlying this developmental process. The ability to visualize the embryonic coronary plexus as a whole, rather than presented in a series of histological sections, is essential to facilitate the analysis of vessel patterning in genetic mutants, and avoids the potential loss of information that can occur as a result of mechanical slicing of the tissue. Vessels fated to form arteries and capillaries are localized deep within the walls of both the ventricles and the aorta^1-3^. However, whilst fluorescent labeling of cells combined with laser-scanning confocal microscopy can provide high resolution images of wholemount-labeled superficial venous/lymphatic vessels^4,5^, the depth of imaging is limited by optical penetration. High-resolution imaging of capillaries and arteries throughout the whole depth of the heart is therefore not possible without some form of tissue clearing.

Poor optical penetration is caused by the high refractive index of the multiple cellular and extracellular (*e.g.,* collagen and elastic fibers) components of thick tissues. This scatters the imaging light, causing blurring and decreased contrast. Clearing agents typically match the high refractive index of such tissues, meaning that light can travel through the sample unobstructed and penetrate deeper into the tissue. Before clearing, tissues are generally dehydrated as water has a relatively low refractive index. A plethora of new clearing methods have been developed recently, however depending on the technique used, the clearing process can take days or weeks and may require costly reagents^6-9^. BABB (a 1:2 mixture of benzyl alcohol and benzyl benzoate) is an inexpensive, commonly used clearing agent, which has the advantage of clearing samples extremely quickly. BABB-based clearing and imaging techniques have been described previously for neurological samples and various organs^10-13^. Here we describe a robust and straightforward technique for the BABB clearance of immunostained samples followed by confocal microscopy, with specific reference to the examination of blood vessels in murine hearts from E (embryonic day) 11.5 - 15.5. However, as has also been demonstrated, the technique can equally well be applied to analysis of whole embryos, as well as other cell types, as long as high quality antibodies to the markers of interest are available.

## Protocol

All animal research was carried out in accordance with institutional guidelines under license from the UK Home Office.

### 1. Dissection and Fixation of Embryonic Hearts

1. Euthanize a timed-pregnant mouse on the required day using an ethically approved culling technique, *e.g., *CO_2_ narcosis followed by cervical dislocation, and remove the embryonic sacs^14^, placing them in a 10 cm Petri dish filled with phosphate buffered saline (PBS).
2. Using a dissecting microscope and forceps, carefully open up the uterine sacs and remove each embryo, severing the umbilical cord and removing the yolk sac.
3. For genotyping purposes, remove the embryonic tail and place in a 1.5 ml microcentrifuge tube for lysis/DNA extraction later.
4. In order to remove the heart, pin each embryo out on its back in a PBS-filled silicone elastomer-coated 35 mm Petri dish, using stainless steel minutien pins. Using fine forceps, carefully open the chest and sever the great vessels, anterior to the heart. Then reaching behind the heart with the forceps, grasp the vessels behind the heart and pull gently to remove the heart; the lungs may also come away at the same time.
5. Transfer the heart to a fresh 35 mm Petri dish containing PBS and use fine forceps to trim away the lung tissue, if necessary. Then transfer the heart to a well of a 48-well plate filled with cold PBS.
6. After collecting all the hearts in the 48-well plate, aspirate the PBS with a fine-tipped plastic Pasteur pipette and replace with 0.5 ml 4% paraformaldehyde (PFA). **CAUTION:** PFA is a known to be allergenic, carcinogenic, and toxic.
  1. Fix E11.5 - 12.5 hearts for 15 min, E13.5 hearts for 20 min and E14.5 - 15.5 hearts for 30 min, in 4% PFA at room temperature.
7. Aspirate the PFA with a fine-tipped plastic Pasteur pipette and rinse the hearts 2x in cold PBS. **CAUTION:** PFA is hazardous and must be disposed of in accordance with institutional regulations.
8. If not using immediately for whole mount immunostaining (see section 2), dehydrate the hearts by carrying out successive 5 min washes in the following solutions: 25% methanol/75% PBS, 50% methanol/50% PBS, 75% methanol/25% PBS. **CAUTION:** Methanol is toxic, wear gloves. Store the hearts at -20 °C in 100% methanol.

### 2. Whole Mount Immunostaining of Embryonic Hearts with Anti-PECAM1 Antibody

NOTE: Anti-PECAM1 antibodies work well for staining the coronary vessels (see step 2.4) but any pan-endothelial marker that gives a robust signal would be suitable.

1. If using hearts that have been stored in 100% methanol, transfer to 1.5 ml microcentrifuge tubes and rehydrate by carrying out successive 5 min washes in the following solutions: 75% methanol/25% PBS, 50% methanol/50% PBS and 25% methanol/75% PBS.
2. Permeabilize the hearts by carrying out 3 x 10 min washes in 0.5 - 1.0 ml PBST (PBS/0.1% Tween-20), rotating at room temperature.
3. Block the hearts by rotating for 1 hr at room temperature in 1.0 ml 10% goat serum in PBST.
4. Remove blocking buffer with a fine-tipped plastic Pasteur pipette or 1,000 μl pipette tip and replace with 400 - 500 μl fresh block containing the anti-PECAM1 primary antibody diluted 1:50. Rotate the tubes overnight at 4 °C.
5. The next day, aspirate the block/primary antibody with a fine-tipped plastic Pasteur pipette or 1,000 μl pipette tip and carry out at least 6x 1 hr washes in 1.0 ml PBST, rotating the tubes at room temperature.
6. Replace the last wash with fresh blocking buffer containing the fluorophore-conjugated secondary antibody diluted 1:500, and incubate overnight at 4 °C, with rotation. Protect from light by wrapping the tubes in foil.
7. The following day, aspirate the block/secondary antibody with a fine-tipped plastic Pasteur pipette and carry out at least 6x 1 hr washes in 1.0 ml PBST, rotating the tubes at room temperature.
8. Store the hearts at 4 °C (protected from light) until needed.

### 3. Preparation of Clearing Solutions and Microscopy Dishes

NOTE: When imaging hearts in BABB it is vital that none of the BABB solution comes into contact with the components of the microscope. For this purpose the following protocol contains instructions for preparing silicone elastomer-sealed dishes suitable for fluorescence microscopy, which can be prepared well in advance. The silicone elastomer provides a barrier to contain the BABB and prevent it from potentially seeping between the glass bottom and the polycarbonate sides of the dish.

1. To prepare the silicone elastomer-coated dishes, take optical glass-bottomed culture dishes and place a cap from a 4 ml screw top vial (approximately 1.5 cm in diameter) in the center of each dish. NOTE: This will form a well for the sample when the elastomer is applied to the dish.
2. Fill the dishes with silicone elastomer to a depth of approximately 0.5 cm, using an applicator cartridge to mix the two components as they are applied, according to the manufacturers' instructions. Leave to cure for at least 24 hr, making sure that the vial cap remains in the center of each dish. NOTE: Use safety glasses to avoid accidental contact of the silicone elastomer with eyes.
3. After curing the elastomer, remove the vial cap from each dish, This will leave a central well where the sample to be imaged can be placed in direct contact with the glass bottom of the dish. NOTE: when cured the elastomer should be firm and dry to the touch.
4. Prepare the BABB solution (1 part benzyl alcohol to 2 parts benzyl benzoate). This should be stored in a glass bottle and protected from light. **CAUTION:** BABB is a toxic, corrosive solution. Handle in a fume cupboard whilst wearing appropriate protective clothing.
5. Mix BABB and methanol to make a 50:50 solution (1 - 5 ml) in a glass vial. Protect from light, *e.g.*, by wrapping the vial in foil.

### 4. Dehydration, Clearing and Mounting of Hearts for Confocal Microscopy

NOTE: Larger samples can be cleared in 4 ml glass vials, however for small samples (*e.g., *hearts up to E13.5) it is better to carry out the clearing process directly in the microscopy dish. This helps prevent 'losing' the samples as the hearts become very transparent once cleared. For these small samples clearing is very rapid, occurring within a few minutes. NOTE: Protect the samples from light during all the following steps by wrapping/covering tubes and dishes with foil.

1. Before clearing, dehydrate the immunostained-hearts through a methanol series by rotating the hearts at room temperature in a 1.5 ml microcentrifuge tube in successive changes of the following solutions: 25% methanol/75% PBS, 50% methanol/50% PBS and 75% methanol/25% PBS (5 min in each). Finally, rotate for 3 x 5 min in 100% methanol.
2. For hearts up to E13.5, place in the center of the microscopy dish; under the microscope ensure the heart is orientated with its dorsal side uppermost. Remove any methanol from around the heart with a fine-tipped plastic Pasteur pipette.
3. Using a clean fine-tipped plastic Pasteur pipette, add BABB:methanol 50:50 solution in a sufficient volume to immerse the heart (approximately 300 - 400 μl). As the hearts can sometimes flip over in the solution, check the orientation again whilst the hearts are still opaque. Leave in the solution for 5 min.
4. Remove the 50:50 solution to a glass waste bottle in the fume hood and replace with BABB, again using a fine-tipped plastic Pasteur pipette. NOTE: A large volume is not required; add sufficient so that the heart sits within a drop of solution. The heart should clear within 5 min.
5. For larger hearts, clear the samples in glass vials. NOTE: An E15.5 heart should clear within 30 min-1 hr but can be left overnight if necessary.
6. After the sample is clear, remove the solution and replace with a minimal volume of BABB. Optionally, cover the sample with a cover slip, to help to keep it in place, however this is not absolutely necessary. Use the heart for imaging.

### 5. Imaging Cleared Hearts by Confocal Microscopy

NOTE: Images were captured on an inverted confocal microscope using 10X or 20X dry objectives. Although it is possible to use the dishes on an upright confocal, extra care must be taken to avoid contact of the objective with the BABB solution.

1. Collect a z-scan of the heart^15^. Depending on the size of the heart, a tile scan may be required to image the whole heart^16^. **CAUTION:** BABB is a corrosive solution, wear clean gloves and ensure the outside of the microscopy dish is free from BABB. Handle the dish carefully and keep the lid on the dish to minimize the risk of accidental spillage onto the microscope.
2. If the objective working distance allows, use a higher magnification to attain higher-resolution pictures of regions of interest.

### 6. Data Analysis Using Fiji Software

1. Open the z stack of images in Fiji^17^.
2. To make a z projection of the whole stack or part of it, click on Image and select Stack, followed by Z project. Enter the Start and Stop slice numbers, and select the type of z projection, *e.g.,* average intensity, max intensity.
3. To highlight individual vessels within a stack of image slices use the Blow tool (from the Plugins menu select Segmentation, and then Blow/Lasso tool) and click on the areas of the image to be filled in each slice. Use the Fill command (click on Edit, then select Fill) to fill the selected areas with the foreground color of choice (click on Edit, then select Options, followed by Colors and Foreground).
4. Z project the image stack as before.

### 7. 3D Surface Volume Rendering of Coronary Arteries Generated Using Imaris

1. Click on the Surpass view icon at the top of the screen and drag and drop the image file into the data window. Click on the 3D View icon at the top of the screen.
2. Select the surfaces icon (blue icon) and then click on the Create tab. Click on the 'Skip automatic creation, edit manually' dialog box and click on the Draw icon (thin pencil icon).
3. Select the Contour tab, and then the Mode tab. In Mode, click on the magic wand or isoline icons. Choose the pointer mode in the Pointer selection on the right hand side of the screen by clicking next to 'Select', and then click on the 'Draw' box (in the bottom left hand corner of the screen).
4. Use the isoline or magic wand tool to select the contours of the arteries in successive slices. Navigate from slice to slice using the Slice Position slider.
5. When all the contours have been selected, click on the Create Surface box. The surrounding volume can be made invisible, or rendered more or less opaque using the Blend mode; click on Volume to highlight it and then select the Settings tab, followed by the Mode tab. Select Blend and use the slider to change the opacity.
6. Capture images using the Snapshot function.

## Representative Results

As the heart develops, the ventricular myocardium is invaded by endothelial cells (ECs) from various sources, and a vascular plexus is formed. Vessels that develop within the ventricular myocardium will go on to form the capillary and arterial networks delivering oxygenated blood to the heart tissue^18^. At the same time (around E11.5) the coronary stems begin to develop independently from a separate capillary network (known as the peritruncal plexus) that surrounds the lumen of the aorta^14,19,20^. Peritruncal plexus ECs initially form multiple connections with the lumen of the aorta at the level of the valve sinuses, however ultimately only one vessel will persist on each side of the aorta, going on to form the roots of the left and right coronary arteries^21^. From around E13.5 the peritruncal and ventricular myocardial vessels join up to form an interconnected network, allowing blood flow from the aorta into the coronary vessels^14,22^. Staining of ECs with anti-PECAM-1 antibody, combined with BABB clearance of the intact hearts, permits the analysis of the developing coronary networks from the earliest possible stages. At later developmental stages the patterning of the maturing coronary arteries can also be examined. This method can also be utilized to stain the vasculature of whole embryos (up to E11.5 at least), and with different antibodies, *e.g.,* anti-alpha smooth muscle actin (Sm22α) and Endomucin.

**Figure 1** shows maximum intensity z-projections of an E11.5 heart generated using Fiji software. The Tile function was used to collect multiple partial images of the heart and stitch them together into a complete image; this is useful to give an overview of staining in the whole specimen. At this stage PECAM1 antibody mainly stains the endocardial lining of the heart and outflow vessels, however a few ECs can also be observed in the left ventricular wall (boxed region in **Figure1A**, shown enlarged in **1A'**). In addition, the peritruncal plexus can be clearly observed in the wall of the outflow tract (OT) (boxed region in **Figure 1B**). In the latter case, reducing the number of slices in the z-stack used to generate the projection improved the clarity of the image, allowing the connections with the lumen of the aorta to be visualized more distinctly (**Figure 1B'**). In **Figure 2**, the increased vasculature proximal to the aorta can be observed in an E12.5 heart. **Figure 3** shows the peritruncal plexus in an E12.5 heart. The 12-slice z projection in **Figure 3A** shows the whole plexus, however as some vessels are localized ventrally to the lumen of the aorta (which is also heavily stained by anti-PECAM1 antibody), splitting the z-stack into a number of sub-stacks (**Figure 3B-D**) provides more visual information. For instance, the sub-stack projected in **Figure 3B** shows a peritruncal vessel connecting to the ventral surface of the aortic lumen, which is not visible in the full projection. **Figure 4** shows part of an E15.5 heart; the coronary arteries are clearly visible at this stage (**Figure 4A**, 30-slice projection). In the 5-slice z projections shown in **Figures 4B** and **C** the aortic and pulmonary valves can also be visualized by PECAM1 staining; the branches and interconnections of the coronary capillary network are also clearer in these smaller sub-stack projections. Manual segmentation techniques have been used to highlight the coronary arteries using Fiji (**Figure 4D**) or Imaris (**Figure 4D** and **E**). The 3D surface volume rendering of coronary arteries/aortic lumen generated using Imaris can be viewed with or without the surrounding vasculature (**Figure 4E** and **F**).

The vasculature of whole embryos can also be examined using PECAM1 staining/BABB clearance. **Figure 4A** and **A'**, show z projections generated from sub-stacks from the right side (z slices 11 - 65) and left side (z slices 66-110) of the embryo respectively. Comparison of the two images shows that the intensity of the fluorescent signal did not significantly decrease with deeper penetration into the tissue. In **Figure 4B**, PECAM1 (red signal) and Endomucin (green signal) antibodies were combined to label the intersomitic arteries (ISAs) and veins respectively, of an E11.5 embryo. **Figure 4C** shows SM22α (red signal) and PECAM1 staining (green signal) of ISAs in an E11.5 embryo. In **Figure 6**, E11.5 embryos stained with PECAM 1 were imaged immediately after BABB clearance (**Figure4A**), or after an interval of approximately two months (**Figure 4B**). The fluorescent signal was still strong enough to image successfully after prolonged storage of the sample in BABB.


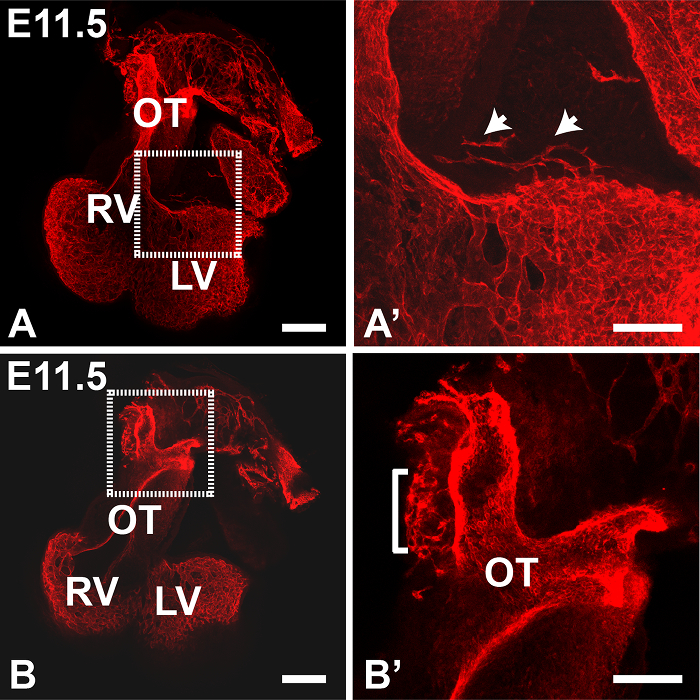
**Figure 1. Immunostaining of an E11.5 Heart with Anti-PECAM1 Antibody (Detected with Anti-rat IgG Conjugated to Alexa 594).** (**A**,**B**) a 2 x 2 tiled image was collected using the 10X objective of an inverted confocal microscope. PECAM1 antibody stains both endocardial and vascular ECs. Projections (maximum intensity) were generated from 22 (**A**) or 5 (**B**) z slices each of 4 μm thickness, using Fiji software. **A' **and **B'** are enlargements of the boxed areas in **A** and **B** respectively. Arrows in **A'** indicate blood vessels in the ventricle wall; in **B'**, the bracket indicates the peritruncal plexus. OT, outflow tract; LV, left ventricle, RV right ventricle. All images are frontal views. Scale bars in A and B = 200 μm; scale bars in A' and B' = 100 μm. Panel 1B' was adapted from Ivins *et al*., 2015 ^19^. Please click here to view a larger version of this figure.


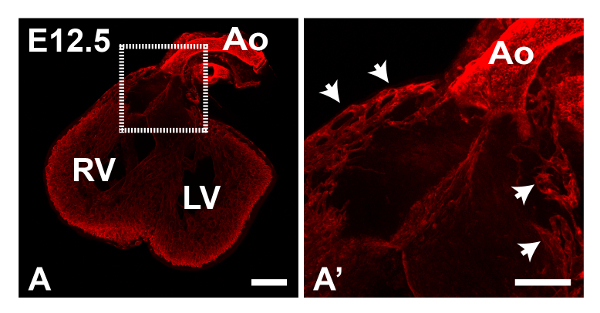
**Figure 2. Immunostaining of an E12.5 Heart with Anti-PECAM1 Antibody. **Panel** A **shows a z projection (maximum intensity) of a 2x2 tiled scan. The boxed region in **A** is shown enlarged in **A'**; arrows indicate multiple blood vessels proximal to the aorta (Ao). LV, left ventricle, RV right ventricle. All images are frontal views. Scale bar in A = 200 μm; scale bar in A' = 100 μm. Please click here to view a larger version of this figure.


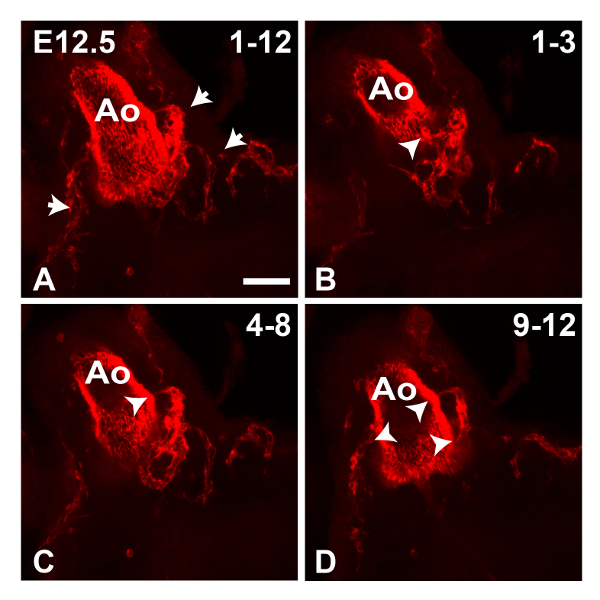
**Figure 3. Imaging of the Peritruncal Plexus in an E12.5 Heart. **Confocal images of an E12.5 aorta (Ao) stained with anti-PECAM1 antibody were collected using the 10X objective. The z projection (maximum intensity) in **A **was generated from 12 z slices (4 μm thickness); arrows indicate the peritruncal plexus vessels. **B-D** The 12 z slices were divided into three sub-stacks as indicated and used to generate separate projections; arrowheads indicate connections formed by the peritruncal plexus to the lumen of the aorta. Peritruncal vessels localized ventrally to the lumen of the aorta are visible in **B **and **C**. All images are frontal views. Scale bar= 50 μm. Please click here to view a larger version of this figure.


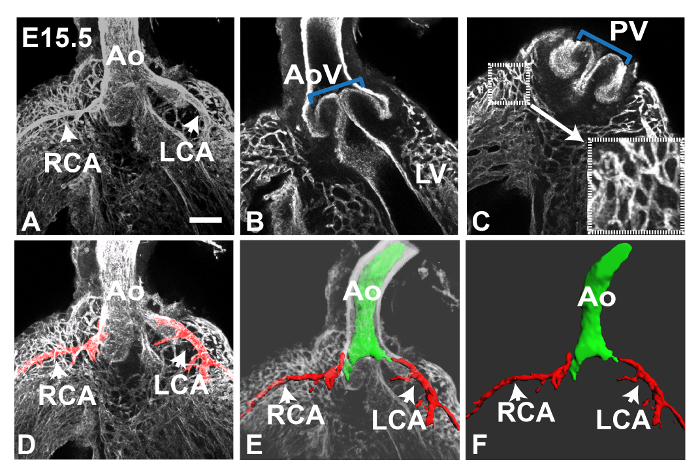
**Figure 4. Coronary Arteries and Capillary Plexus in an E15.5 Heart.** Confocal images of an E15.5 heart stained with anti-PECAM1 antibody were collected using the 10X objective. Panel **A** shows a maximum intensity z projection generated from 30 z slices (4 μm thickness); arrows indicate the left and right coronary arteries (LCA and RCA) which can be seen connecting to the aorta (Ao). Using different 5 z slice sub-stacks the aortic and pulmonary valves (AoV and PV) can be seen in **B** and **C** respectively (blue brackets). The ventricular capillary plexus is also clearer in these smaller sub-stack projections; blood vessels proximal to the pulmonary valve (small box) are shown enlarged in the bottom right of panel **C **(large box). Fiji was used to highlight individual blood vessels, *e.g.*, for panel **D** the Blow/lasso tool was used to fill the coronary arteries with red color manually in each z slice prior to projection. Imaris software was used to create 3D images of the arteries (red) and aortic lumen (green) in **E **and **F**; again this was done manually, using the magic wand tool to create object contours in each z slice. The opacity of the surrounding volume can be altered in Blend mode, as in **E**. Alternatively, the 3D image can be extracted, as shown in **F**. LV, left ventricle**. **All images are frontal views. Scale bar in A = 100 μm. Please click here to view a larger version of this figure.


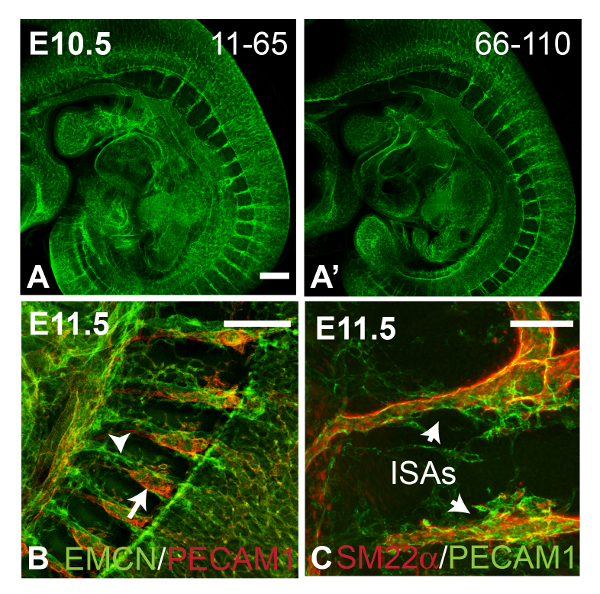
**Figure 5. Imaging of Embryonic Vasculature.** Panels **A** and **A'** show confocal images of an E10.5 wild type embryo stained with anti-PECAM1 antibody, collected using the 10X objective (tiled scan). Average intensity z projections were generated from z slices 11-65 (**A**) and 66-110 (**A'**) of a 120 z slice scan (4 μm thick slices), showing only a slight loss of fluorescence intensity across the thickness of the embryo. Panel **B** shows the intersomitic vessels of an E11.5 embryo stained with antibodies against PECAM1 (red signal from 594-conjugated secondary antibody) and Endomucin (EMCN, green signal from 488-conjugated secondary antibody), which stain the intersomitic arteries (arrow) and veins (arrowhead) respectively (average intensity z projection from a tiled scan). Panel **C** shows intersomitic arteries (ISAs) from an E11.5 wild type embryo stained with antibodies against PECAM1 (green signal from 488-conjugated secondary antibody) and SM22α (red signal from 594-conjugated secondary antibody). Confocal images were collected using the 20X dry objective and used to generate maximum intensity z projections. All images are sagittal views. Scale bars in A and B = 250 μm, scale bar in C = 100 μm. Please click here to view a larger version of this figure.


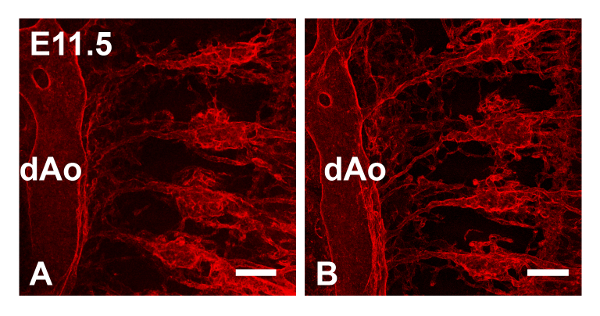
**Figure 6. Immunostained Samples Cleared in BABB Retain Fluorescent Signal for at Least two Months.** E11.5 embryos were stained with PECAM1 antibody and cleared with BABB; embryos from the same batch were imaged immediately or after an interval of two months. Panel **A** shows the intersomitic arteries of the embryo imaged immediately and panel **B** shows the embryo imaged two months later. Confocal images were collected using the 10X dry objective and used to generate maximum intensity z projections. dAO, dorsal aorta. Images show sagittal views. Scale bars in A and B = 100 μm. Please click here to view a larger version of this figure.

## Discussion

Coronary vessels in whole embryonic hearts were imaged by wholemount immunostaining with anti-PECAM1 antibody followed by optical clearance and confocal microscopy. The straightforward method described here, for the clearance of embryonic mouse hearts with BABB, increases optical penetration and enables the capture of high-resolution images of blood vessels localized in the aorta and ventricular walls. Glycerol-based mounting reagents such as Vectashield (refractive index 1.45) have also been used for imaging of the coronary vasculature^22^ however the higher refractive index of BABB (1.56) reduces light scattering still further, enabling deeper tissue penetration. Tissue clearance obviates the need for more complex, expensive forms of microscopy such as multiphoton and light sheet microscopy which may be less readily available to researchers. The clearing process is extremely rapid compared to other methods^6-9^ and for small samples can be carried out using small volumes of reagents directly on the microscopy dish. Robust staining of the vasculature is required in order to achieve high quality images; anti-PECAM1 antibody was selected as it marks all types of coronary ECs and a number of commercial antibodies were found to give suitably high levels of staining. In addition, the fluorescent staining appears to be extremely stable in BABB; samples stored at room temperature in BABB (protected from light) retained their fluorescent signal for several months. The fact that PECAM1 antibody efficiently labels the coronary endocardium as well as the vasculature was occasionally problematic, especially when capturing images of the peritruncal plexus. Stronger staining of the aortic lumen compared to the peritruncal ECs resulted in a risk of over-saturation in some areas of the images, meaning that careful adjustment of the imaging parameters was required. Ideally, an antibody staining only vascular ECs would be used; in practice, however, finding suitable antibodies that yield the required level of wholemount staining can be difficult. Recently, fatty acid binding protein 4 (FABP4) has been shown to be a marker of coronary vascular ECs^23^ and may therefore represent an alternative to PECAM1.

In order to retain the 3D morphology of the aorta and heart chambers the samples were not flat-mounted, but were instead imaged in glass-bottomed dishes. The depth of the samples to be imaged precluded the use of high magnification objectives, due to their short working distances. However high-resolution images were still achievable using a 10X objective by increasing pixel dwell time and using a pixel array size of at least 1,024 x 1,024 for image capture. This was sufficient for the analysis of the structure and distribution of coronary vessels, however finer analysis of cellular structure may require flat-mounting of samples. Dissection of individual parts of the heart for mounting, *e.g., *ventricle walls, or aorta, may also be necessary. Alternatively, over-sampling of the image followed by deconvolution may be carried out in order to increase resolution and sensitivity; this however requires considerably longer scanning times and creates extremely large image files that require a lot of computing power to process.

Hearts up to E15.5 were successfully imaged using this method, and it is also possible to analyze the vasculature of whole embryos (up to at least E11.5) using the same protocol. Other cell types,* e.g., *smooth muscle cells have also been imaged in our laboratory using this technique. For thicker tissues, *e.g.,* hearts older than E15.5, antibody penetration can be a limiting factor; longer incubations and/or increased detergent may be required. In addition, when collecting a large z stack of images signal strength can be reduced as the lasers penetrate the furthest portions of tissue; however the confocal settings can be adjusted to increase laser power with increasing z distance.

This method facilitates confocal microscopic analysis of both the earliest stages of coronary vessel formation and the patterning of the coronary arteries at later developmental stages. Detailed information about the distribution, branching and structure of blood vessels can be acquired in a short time, making this a valuable tool for the study of genetic mouse mutants with specific defects in angiogenesis pathways.

## Disclosures

The authors have nothing to disclose.
